# *Arabidopsis* senescence-associated protein DMP1 is involved in membrane remodeling of the ER and tonoplast

**DOI:** 10.1186/1471-2229-12-54

**Published:** 2012-04-24

**Authors:** Alexis Kasaras, Michael Melzer, Reinhard Kunze

**Affiliations:** 1 Dahlem Centre of Plant Sciences (DCPS), Freie Universität Berlin, Institut für Biologie - Angewandte Genetik, Albrecht-Thaer-Weg 6, D-14195, Berlin, Germany; 2 Institute of Plant Genetics and Crop Plant Research (IPK), Corrensstraße 3, D-06466, Gatersleben, Germany

## Abstract

**Background:**

*Arabidopsis* DMP1 was discovered in a genome-wide screen for senescence-associated membrane proteins. DMP1 is a member of a novel plant-specific membrane protein family of unknown function. In rosette leaves *DMP1* expression increases from very low background level several 100fold during senescence progression.

**Results:**

Expression of AtDMP1 fused to eGFP in *Nicotiana benthamiana* triggers a complex process of succeeding membrane remodeling events affecting the structure of the endoplasmic reticulum (ER) and the vacuole. Induction of spherical structures (“bulbs”), changes in the architecture of the ER from tubular to cisternal elements, expansion of smooth ER, formation of crystalloid ER, and emergence of vacuolar membrane sheets and foamy membrane structures inside the vacuole are proceeding in this order. In some cells it can be observed that the process culminates in cell death after breakdown of the entire ER network and the vacuole. The integrity of the plasma membrane, nucleus and Golgi vesicles are retained until this stage. In *Arabidopsis thaliana* plants expressing AtDMP1-eGFP by the 35S promoter massive ER and vacuole vesiculation is observed during the latest steps of leaf senescence, whereas earlier in development ER and vacuole morphology are not perturbed. Expression by the native *DMP1* promoter visualizes formation of aggregates termed “boluses” in the ER membranes and vesiculation of the entire ER network, which precedes disintegration of the central vacuole during the latest stage of senescence in siliques, rosette and cauline leaves and in darkened rosette leaves. In roots tips, *DMP1* is strongly expressed in the cortex undergoing vacuole biogenesis.

**Conclusions:**

Our data suggest that DMP1 is directly or indirectly involved in membrane fission during breakdown of the ER and the tonoplast during leaf senescence and in membrane fusion during vacuole biogenesis in roots. We propose that these properties of DMP1, exacerbated by transient overexpression, may cause or contribute to the dramatic membrane remodeling events which lead to cell death in infiltrated tobacco leaves.

## Background

DMP1 (DUF679 Membrane Protein 1) is a short membrane protein of 207 amino acids with four transmembrane spans and belongs to a small, strictly plant-specific protein family comprising ten members in *Arabidopsis thaliana*[1]. *DMP1* is transcriptionally up-regulated during developmental senescence (NS) in siliques, rosette and cauline leaves, during dark induced senescence in attached (DIS) and detached leaves (DET) and is expressed in the phloem bundles of roots and the cortex of root tips [2]. In all three senescence programs, *DMP1* expression increases from the onset until the very late stages of senescence. This suggests conserved functions during developmental and induced senescence as well as an involvement during the entire senescence program. *DMP1* is also expressed in the dehiscence and abscission zones of siliques [1], which indicates a role in programmed cell death (PCD).

In metazoans, based on cell morphology apoptosis, autophagy and necrosis are distinguished as the three main PCD forms. In plants “autolytic” and “non-autolytic” PCD are differentiated [3]. Non-autolytic PCD is marked by the absence of rapid cytoplasm clearance [3], as is observed e.g. in hypersensitive response and endosperm degeneration. Autolytic PCD is characterized by rupture of the tonoplast and subsequent rapid cytoplasm clearance and occurs e.g. in tracheary element differentiation and senescence, although the relationship between senescence and PCD is still controversial [4-6]. In the present study, we use the term PCD for the terminal stage of leaf senescence. The earliest detectable alterations during leaf senescence are changes in the ultrastructure of chloroplasts. In the course of senescence all organelles are eventually degraded. In *Iris* and carnation petal senescence, ER and attached ribosomes, Golgi bodies and mitochondria have been reported to be degraded during senescence before vacuolar collapse [7]. Ultrastructural, biochemical and gene expression data indicate that large-scale autophagy is involved in these degradation processes [8]. However, the fate of organelles has been almost exclusively investigated by electron microscopy using fixed cells. Investigations of ultrastructural changes of organelles undergoing senescence using fluorescence tags in living cells are scarce.

Here we present an extensive characterization of the complex cellular processes induced by the senescence-associated DMP1 protein fused to eGFP in *Nicotiana benthamiana* and *Arabidopsis thaliana* by confocal fluorescence and electron microscopy. In tobacco, DMP1-eGFP overexpression triggers membrane remodeling, expansion, fusion and fission events at the tonoplast and the ER. We classified the successive remodeling events into five stages and showed that they ultimately can lead to cell death by extensive fragmentation of the ER and the vacuole. We note the formation of an additional network that we propose to be proliferating smooth ER. To our knowledge, this is the first observation of a clear separation of rough and smooth ER of the cortical ER in tobacco using fluorescent tags. Thus, overexpression of DMP1-eGFP might induce a differentiation of the cortical ER. In *Arabidopsis* we investigated DMP1-eGFP fluorescence patterns in tissues undergoing NS or DIS as well as the response to whole plant darkening, a treatment that induces a range of physiological effects which are not related to NS and DIS [9]. We found that in all tissues and senescence types DMP1-eGFP illuminates vesiculation events of the ER and the tonoplast and the formation of aggregates (“boluses”) within the ER. The formation of boluses, which suggest altered protein flow and the vesiculation of the entire ER network, has not been reported during senescence yet. We suggest that rupture of the tonoplast, a hallmark of autolytic PCD in the terminal senescence stage, may be accompanied or preceded by fragmentation of the vacuole. The effects of DMP1-eGFP expression in tobacco and *Arabidopsis* suggest that DMP1 regulates membrane folding and is involved in tonoplast and ER membrane fusion and fission reactions.

## Results

### Expression of DMP1-eGFP in *Nicotiana benthamiana* epidermis cells induces membrane remodeling

**Figure 1:**
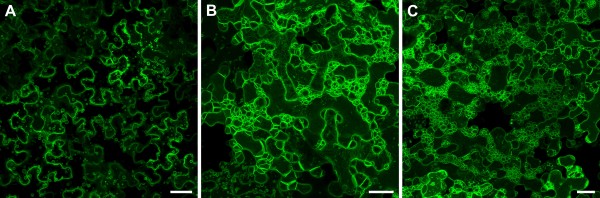
**Temporal dynamics of DMP1-eGFP fluorescence patterns in tobacco epidermis cells.** Representative overviews of tobacco epidermis cells expressing DMP1-eGFP at 2 dpi (**a**), 5 dpi (**b**) and 7 dpi (**c**). Scale bar, 40 μm.

To investigate intracellular targeting of DMP1 we agroinfiltrated a *35S:DMP1-eGFP* construct into tobacco leaves. Interestingly, the fusion protein displayed a highly dynamic and temporally changing fluorescence pattern (Figure 1). Two to three days post infiltration (dpi), the first fluorescence signals became visible and labeled the boundaries of the cells and spherical structures inside the lumen of the central vacuole (Figure 1a). Until five dpi the fluorescence pattern changed and the cells underwent membrane remodeling to various degrees (Figure 1b). Two days later the majority of cells exhibited severely remodeled endomembranes, giving the cells a “foamy” appearance (Figure 1c). These membrane remodeling patterns and time courses were highly reproducible with only little fluctuation in severity. To investigate whether the observed membrane remodeling events are due to overexpression of DMP1-eGFP by the strong, constitutive 35S promoter, we also expressed the protein by the endogenous, senescence-specific *DMP1* promoter. This promoter led to somewhat weaker expression levels in tobacco and induced a somewhat weaker membrane remodeling phenotype, that was reproducible and comparable to the 35S promoter-induced phenotype though. Thus, the observed membrane remodeling patterns are not merely overexpression artifacts.

**Figure 2:**
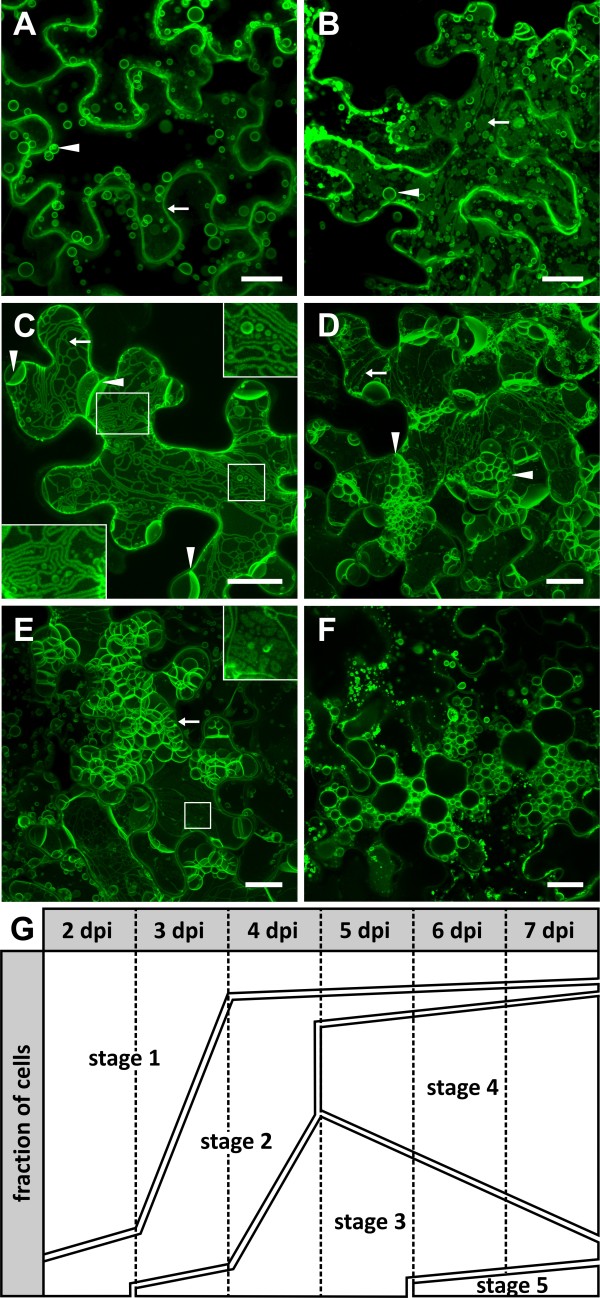
**DMP1-eGFP induces membrane remodeling in*Nicotiana benthamiana.*** Transient overexpression of DMP1-eGFP in tobacco epidermis cells results in distinct fluorescence patterns classified into five stages: stage 1 (**a**), stage 2 (**b**), stage 3 (**c**), stage 3 to stage 4 transition (**d**), stage 4 (**e**) and stage 5 (**f**). (**g**) Schematic of the dynamic alteration in DMP1-eGFP fluorescence patterns from stage 1 to stage 5. Scale bar, 20 μm.

We classified the course of endomembrane remodeling into five stages. Stage 1 is characterized by well-defined fluorescence signals along the cell walls (Figure 2a, arrow) and at spherical structures located inside the lumen of the vacuole (Figure 2a, arrowhead). Three to four dpi the cells typically enter stage 2 where they begin to display extended membrane sheets within the cytoplasm reminiscent of ER cisternae (Figure 2b, arrow) and bulbs (Figure 2b, arrowhead). Stage 3 is distinguished by large tubular and reticulated structures forming a network reminiscent of cortical ER (Figure 2c, arrow). Also spherical bodies are visible (Figure 2c, insets), but unlike the spherical structures in stages 1 and 2 they appear to be located in the cytoplasm, and large membrane sheets crossing and thereby compartmentalizing the central vacuole emerge (Figure 2c, arrowheads). Figure 2d shows a cell in transition from stage 3 with its distinctive tubular structures (Figure 2d, arrow) to stage 4 with its typical “foamy” membrane meshwork (Figure 2d, arrowheads). In stage 4 a great deal of the central vacuole is filled with this “foamy” membrane mesh (Figure 2e, arrow). Some residual tubular structures are still present, and occasionally enigmatic, sponge-like structures appear (Figure 2e, inset). In the terminal stage 5 the vacuole breaks down by vesiculation (Figure 2f). This stage was rarely observed because the cells appear to die rapidly after vacuole disintegration and only a minor fraction of stage 4 cells enter stage 5. Remarkably, in spite of strong membrane remodeling the cells seem to stay viable for a prolonged period of time without entering vesiculation. Figure 2g shows the approximate fractions of cells in stages 1 to 5 at different times after infiltration.

To characterize the membrane structures labeled by DMP1-eGFP we subsequently performed colocalization experiments with various membrane markers.

### Stage 1: The tonoplast located DMP1-eGFP induces the formation of bulbs

**Figure 3:**
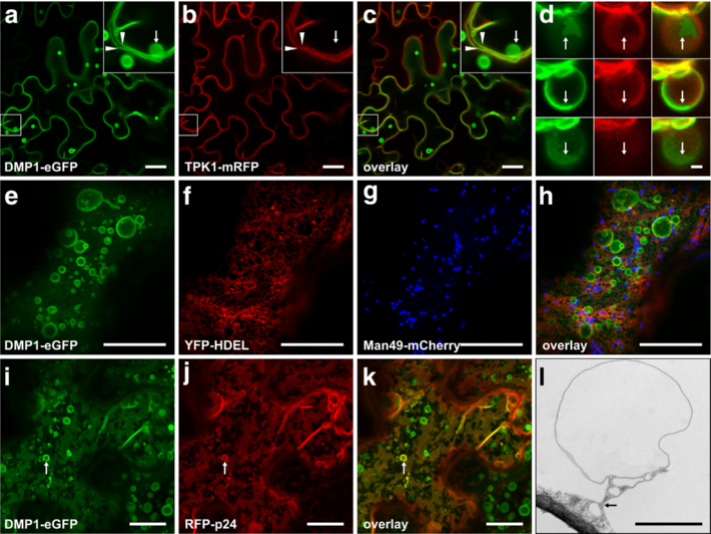
**Stages 1 and 2.** Co-expression of DMP1-eGFP (**a**) and the tonoplast marker TPK1-mRFP (**b**) shows in stage 1 colocalization at the vacuolar membrane (**c**) and occasional partial overlap at bulbs (**d**). Co-expression in stage 1 of DMP1-eGFP (**e**), YFP-HDEL labeling the ER lumen (**f**) and Man49-mCherry decorating Golgi vesicles (**g**) shows no localization of DMP1-eGFP in Golgi vesicles and no or only weak signals in the ER (**h**). Co-expression during stage 2 of DMP1-eGFP (**i**) and the ER membrane marker RFP-p24 (**j**) shows colocalization in the ER that exhibits cisternal morphology (**k**). Bulbs in tobacco epidermis cells visualized by electron microscopy (**l**). Scale bar, 20 μm except D, 2 μm.

The first DMP1-eGFP fluorescence signals were observed at the cell periphery and in spherical structures two days after infiltration (Figure 3a). Upon co-infiltration DMP1-eGFP clearly colocalized with the tonoplast marker TPK1-mRFP (Figure 3b,c, arrowheads), but not with the plasma membrane marker mRFP-MUB2 (data not shown). TPK1-mRFP was largely excluded from the spherical structures (Figure 3b,c, arrows) which supposedly are identical to the “bulbs” reported by Saito and colleagues [10] as they are comparable in size, motility and fluorescence intensity. Overlap between DMP1-eGFP and TPK1-mRFP fluorescence at the bulbs was extremely rare and only partial. Some regions of the bulbs were labeled with either DMP1-eGFP or TPK1-mRFP (Figure 3d, arrows), suggesting different membrane properties and rapid exclusion of TPK1-mRFP from the bulbs. As γ-TIP-mCherry did not lead to proper fluorescence signals in tobacco [1] it could not be used as an alternative tonoplast/bulb marker. We therefore studied DMP1-eGFP infiltrated tobacco leaf epidermis cells by transmission electron microscopy. In DMP1-eGFP expressing epidermis cells we observed a significantly higher number of bulbs (Figure 3l) than in mock-transformed cells, supporting the notion DMP1-eGFP induces formation of these bulbs. DMP1-eGFP was never observed in Golgi vesicles (Figure 3e,g,h) and was largely excluded from the ER (Figure 3e,f,h) which had a normal tubular morphology. The same result was obtained by using the integral fusion protein RFP-p24 instead of the luminal YFP-HDEL as ER marker ( Additional file 1: Figure S1).

### Stage 2: Reorganization of the ER - transition from tubular elements to cisternae

Stage 2 is characterized by the appearance of bulky cisternae in the cytoplasm that strongly resemble cortical ER observed under certain conditions (see Discussion), while the bulbs and tonoplast labeling from stage 1 are still retained (Figure 1b). The ER localization of DMP1-eGFP was verified by co-expression with RFP-p24 (Figure 3i,j,k). We also occasionally observed RFP-p24 signals in bulbs (Figure 3j,k, arrows). This might either indicate mislocalization of the ER marker due to overexpression or some dysfunction of the ER during stage 2.

### Stage 3: De novo formation of a cortical ER-derived network inside the cytoplasm and vacuolar sheets inside the vacuole

**Figure 4:**
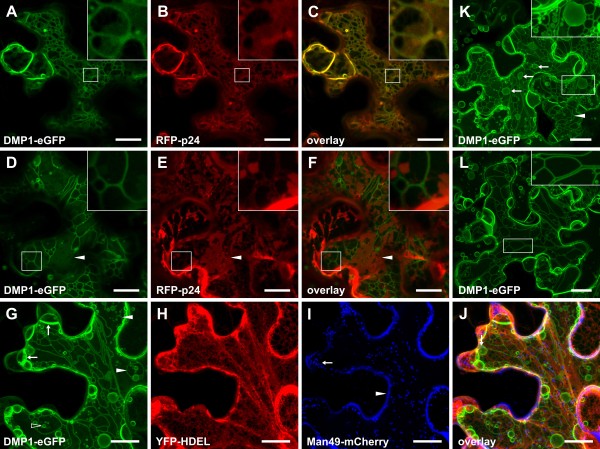
**Stage 3**. Co-expression of DMP1-eGFP and ER markers during stage 3 shows cells with partial colocalization (**a-c and d-f**) and cells lacking colocalization (**g-j**). Accordingly, in cells expressing DMP1-eGFP alone, ER with cisternal morphology can be distinguished in the background in some of them (**k**) but not in others (**l**). Co-expression of DMP1-eGFP (**a**) and RFP-p24 (**b**) shows colocalization with the ER network (**c**) except for tubular structures where RFP-p24 is excluded (insets **a-c**). Tubular network dissociating from the ER (**d-f**). Weak residual DMP1-eGFP signals in the cisternal ER network (**d-f**, arrows). Vacuolar sheet formation inside the vacuole occurs preferentially at the periphery of cells (**g**, arrows) that still exhibit a DMP1-labeled tubular network and cytosolic vesicles which are either connected to this network (**K**, inset) or suspended in the cytosol (**k**, inset, and **g**, arrow). Tubules are often closely spaced but unconnected (**k**, inset). DMP1-eGFP (**g**) and YFP-HDEL (**h**, false-colored) do not colocalize (**j**) and Golgi vesicles labeled by Man49-mCherry are intact (I false-colored, arrow). Man49-mCherry accumulates to some extent in the apoplast (**i**, arrowhead). Scale bar, 20 μm.

Stage 3 is marked by different membrane remodeling events. Most conspicuously is the labeling of novel tubular structures which do not colocalize with the different markers used. In stage 3 DMP1-eGFP and RFP-p24 both decorate the whole ER network composed principally of cisternae (Figure 4a,b,c). DMP1-eGFP additionally decorates another tubular mesh from which RFP-p24 is excluded (Figure 4a,b,c, insets). However, both networks share the same overall pattern, indicating either physical connection or differential labeling of the same entity. Strikingly, over time DMP1-eGFP and RFP-p24 progressively segregate. While DMP1-eGFP initially colocalizes with RFP-p24 in the ER cisternae ( 4d,e,f, arrowhead), the tubular structures mostly dissociate from the ER network (Figure 4d,e, f, insets). In late stage 3, when first vacuolar sheets and “foamy” structures emerge (Figure 2c and 4g, arrows), DMP1-eGFP is almost undetectable in the ER network labeled by YFP-HDEL (Figure 4g,h). This time course suggests that the tubular structures derive directly from the ER and coincide with a progressive exclusion of DMP1-eGFP from the ER. The tubules labeled only by DMP1-eGFP form an interconnected network throughout the cytoplasm (Figure 4a,g,k and l), are homogeneous in diameter and show a smooth and relaxed appearance (Figure 4a,d,g,k,l and 2c), and are - in contrast to the repetitive polygonal structure of the cortical ER network - often tightly packed and peculiarly folded (Figure 2c, insets and 4 l, inset). Large swollen spherical formations reminiscent of ER cisternae are often observed at the intersection of DMP1-eGFP-labeled tubules (Figure 4g and k, arrows and inset). In late stage 3, isolated tubules are also found (Figure 4g, arrowheads and K, arrowhead) whose occurrence coincides with the presence of cytosol-located vesicles (Figure 4g, empty arrowhead, k, inset and l). These vesicles and the isolated tubules likely derive from the DMP1-eGFP-labeled network by fission events.

As mentioned above, vacuolar sheets crossing the lumen of the vacuole and first “foamy” membranes appear in stage 3 and accumulate gradually (Figure 2c). The density of vacuolar sheets correlates with a progressive loss of the DMP1-eGFP labeled network. Moreover, the tubules were occasionally found tightly associated with these vacuolar sheets (Additional file 1: Figure S2). These observations suggest a connection between these two structures. Golgi vesicles appeared to be unaffected during stage 3 (Figure 4i) suggesting proper ER-Golgi transport despite extensive remodeling of the ER.

### Stage 4: Formation of “foamy” membrane structures inside the vacuole

**Figure 5:**
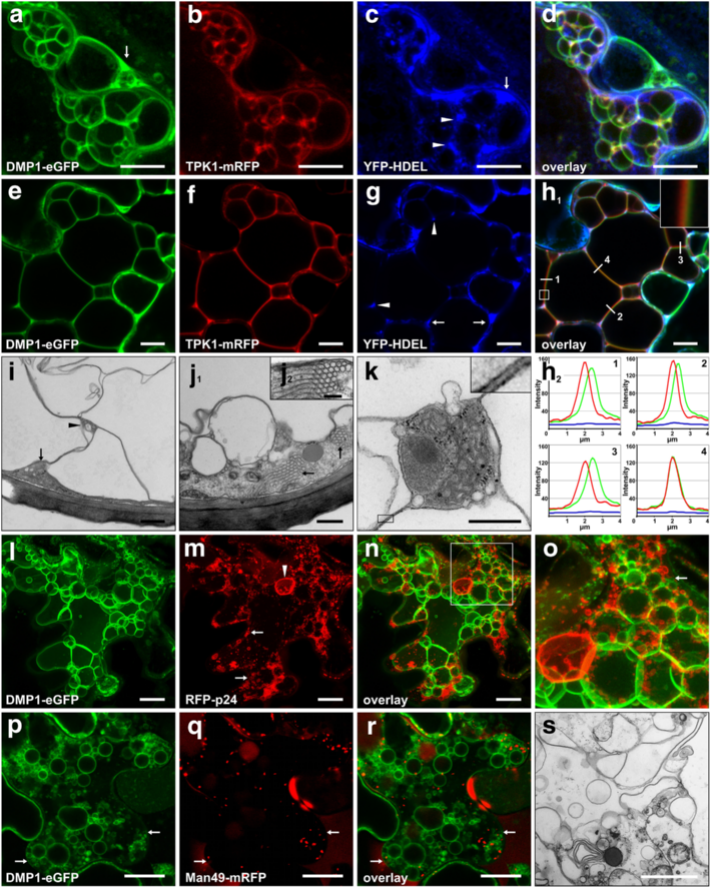
**Stages 4 and 5.** Co-expression of DMP1-eGFP (**a**), TPK1-mRFP (**b**) and YFP-HDEL (**C**, false-colored) shows colocalization of DMP1-eGFP and TPK1-mRFP and dissociation from the ER (**d**). The ER is compressed into interstices and junctions formed by foamy membrane structures (**c** and **g**, arrows and arrowheads respectively). On single plane images (**e-h**_1_) but not maximum projection (**a-d**), the colocalization between DMP1-eGFP (**e**) and TPK1-mRFP (**f**) appears incomplete (**h**_1_). In the majority of membrane segments analysed the fluorescence signal peaks are shifted between 200 nm and 300 nm (**h**_**2**_, panels 1–3), suggesting a double membrane structure, one membrane being labeled by DMP1-eGFP and the other with TPK1-mRFP. In some membrane segments the fluorescence peaks match perfectly, indicating colocalization (**h**_1_ and **h**_2_, segment and panel 4 respectively). Vacuolar sheets and foamy membrane formations have double membranes (**i** and **k**). Interstices and membrane junctions contain cytoplasm and trapped organelles (**i**, arrow and arrowhead). Crystalloid ER in a late stage 4 cell (**j**_1_ and **j**_2_). Cells displaying foamy vacuolar membrane structures (**l**) and labeling of the whole tonoplast (**p**) enter cell death by vesiculation of the entire ER network (**m**, arrows) except for the nuclear envelope (**m**, arrowhead). DMP1-eGFP (**l**) and RFP-p24 (**m**) were fully dissociated (**n**, magnified in **o**) as in stage 4. The Golgi vesicles remain unaffected in these cells (**q** and **r**). This process was visualized by EM (**s**). Scale bar, A-H_1_ and S, 10 μm; I, J_1_ and K, 0,5 μm; L-N and P-R, 20 μm; J_2_, 0,1 μm.

Transition from stage 3 to 4 is indicated by the appearance of “foamy” membrane formations that coincide with a decrease in tubular structures (Figure 2d). The “foamy” membranes likely derive from accumulation of vacuolar sheets. At this time no DMP1-eGFP signals are detected in the ER anymore (Figure 5a,c,d and e,g,h_1_) which appears to be compressed into interstices (Figure 5c, g, arrows) and junctions of the “foamy” membranes (Figure 5c,g, arrowheads). The junctions contain different organelles such as peroxysomes or mitochondria (Figure 5i, arrowhead and k) as found in transvacuolar strands [11]. Confocal fluorescence microscopy (Figure 5h_1_) and electron microscopy (Figure 5i,j,k) consistently revealed that the vacuolar sheets and “foamy” membranes are double membranes. DMP1-eGFP (Figure 5e) and TPK1-mRFP (Figure 5f) do not perfectly colocalize as shown by separation of the two fluorescence signals (Figure 5 h_1_ and h_2_). The distance between the two fluorescence peaks is about 200 nm to 300 nm (Figure 5 h_1_ and 5 h_2_ membrane segments 1, 2 and 3) which would allow small organelles to pass through. The double-membrane topology is corroborated by the observation of ER squeezed between the two membranes of a membrane sheet (Additional file 1: Figure S3). Occasionally however, perfect colocalization is observed which might indicate localization of both fusion proteins at both membranes (Figure 5 h_1_ and 5 h_2_ membrane segment 4). Under electron microscopy the double membranes appear more closely stacked (Figure 5i and k). However, this may be a fixation artefact and not reflect the situation *in vivo*. Membrane sheets consisting of a single membrane were never observed by EM. In 70 nm thin cross-sections the double membranes completely crossed the lumen of the vacuole, confirming that they correspond to the vacuolar sheets and not to transvacuolar strands (TVS) as the latter are unlikely straight and oriented in parallel to the section cut across the whole vacuole. TPK1-mRFP is often excluded from regions within foamy membrane structures (Additional file 1: Figure S4). Interestingly, these areas are located at contact zones between adjacent sheets within foamy membrane structures.

During stage 4 intriguing sponge-like flat structures arise (Figure 2e, inset, Additional file 1: Figure S5). TPK1-mRFP is excluded from these areas (Additional file 1: Figure S5) which is reminiscent of the observations in individual bulbs (Figure 3d) and within foamy structures (Additional file 1: Figure S4). We hypothesize that these sponge-like structures represent residual TPK1-mRFP-free membrane domains derived from bulbs and vacuolar sheets. Additionally we observed the formation of crystalloid ER (Figure 5 j_1_,j_2_).

### Stage 5: Vesiculation of the vacuole and the ER leading to cell death

Six days post infiltration some cells with severe vesiculation of endomembranes also display overall intracellular disintegration, indicating the onset of cell death (Figure 2f). As in stage 4, DMP1-eGFP only labels the tonoplast and foamy membrane formations but not the ER (Figure 5l,m,n). The ER is not reticulated but highly vesiculated (Figure 5m,n,o, arrow). The vacuolar and foamy membranes also appear to vesiculate more heavily than in stage 4 and form smaller vesicles (Figure 5o, arrow and p). Despite the obvious breakdown of the ER, the integrity of the nuclear membrane (Figure 5m, arrowhead and o) and Golgi vesicles (Figure 5q) is still retained. The Golgi marker, which is partially secreted to the apoplast ( 4i), indirectly indicates in Figure 5p that the plasma membrane, not labeled by DMP1-eGFP, is still intact (Figure 5p,q,r, arrows). The massive vesiculation of endomembranes was confirmed by electron microscopy (Figure 5s).

### Expression of DMP1-eGFP in transgenic *Arabidopsis thaliana* reveals dual ER/tonoplast localization

**Figure 6:**
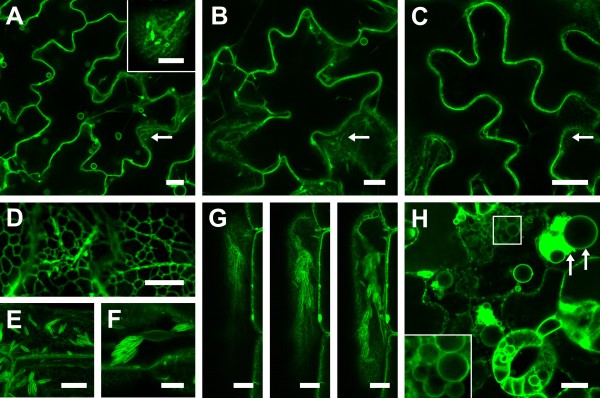
**Dual ER/tonoplast localization of DMP1-eGFP in stably transformed*A. thaliana plants.*** Overexpression of DMP1-eGFP in in young emerging Arabidopsis cotyledons (7 DAS) leads to labeling of the tonoplast and bulbs (**a**), the ER network (**a**, arrow and inset) and ER bodies (**a**, inset). In 12 DAS cotyledons the number of bulbs decreases (**b**) and at 18 DAS bulbs are no longer visible (**c**). In rosette leaves intense ER labeling is observed (**d**). In hypocotyls the ER is associated with single ER bodies (**c**), with ER body clusters (**f**), or with large ER body aggregates extending across the whole cell (**g**). Massive vesiculation of endomembranes occurs during dark induced senescence (**h**). Scale bar, 10 μm.

As dual tonoplast/ER localization of a protein is unusual we aimed to determine if dual tonoplast/ER localization and induction of membrane remodeling by DMP1 overexpression is conserved in transgenic plants. In *Arabidopsis* plants carrying a *35S:DMP1-eGFP* transgene, seven days after sowing (DAS), bulbs and tonoplast localization is observed in young cotyledons (Figure 6a). Five days later (12 DAS) the number of bulbs decreases (Figure 6b) and at 18 DAS no more bulbs were visible (Figure 6c). This time course of bulb development is consistent with previous observations using γ-TIP as marker [10]. In addition to accumulation in bulbs strong DMP1-eGFP signals are observed in the ER as well as in ER bodies in all these stages (Figure 6a, b, c, arrows and inset). The ER bodies vanish as the cotyledons age, corroborating earlier reports [12]. Accordingly, in cotyledons of *Arabidopsis* DMP1-eGFP is dually targeted to the ER and the tonoplast, but overexpression of DMP1-eGFP does not affect the morphology and development of the ER and the tonoplast in this organ. ER bodies are also labeled by DMP1-eGFP in hypocotyl cells somewhat later in development (Figure 6g). In rosette leaves, we observe an intense, leaf-age independent accumulation of DMP1-eGFP in the ER (Figure 6d). In addition, protoplasts prepared from rosette leaves also show some tonoplast localization, confirming the dual localization seen in cotyledons (data not shown). During developmental leaf senescence and even more pronounced during dark induced leaf senescence (Figure 6h), individual cells or leaf areas show massive vesiculation reminiscent of the cellular breakdown process during stage 5 in tobacco. Thus, in *Arabidopsis* leaves DMP1-eGFP appears to be similarly associated in disintegration of the ER and the vacuole by vesiculation as in tobacco (Figure 6h, arrows).

### Expression of DMP1-eGFP from the DMP1 promoter in *Arabidopsis* highlights formation of boluses within the ER and fragmentation of ER and tonoplast during senescence

**Figure 7:**
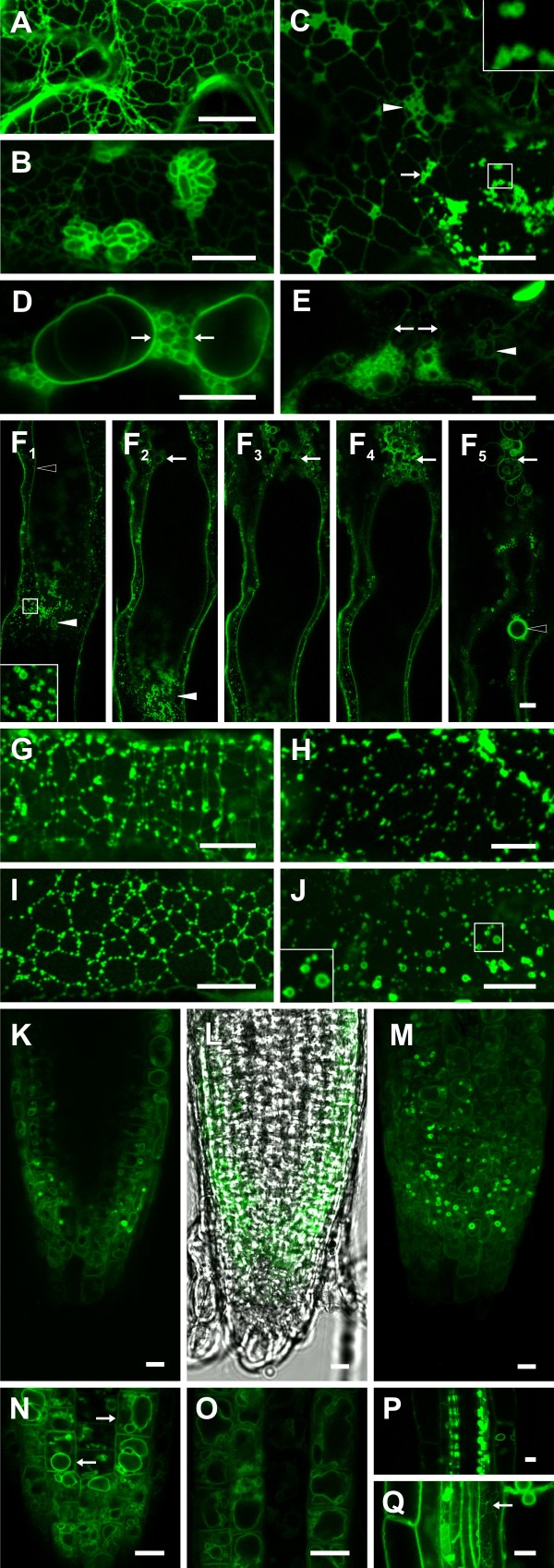
**DMP1-eGFP fluorescence patterns during development in*A. thaliana.*** In mature/early senescing rosette leaves DMP1-eGFP expressed from the native DMP1 promoter localizes in the ER (**a**) and occasionally in ER bodies (**b**). Vesiculation of the ER in rosette leaves during late NS (**c**). Vesiculation of the vacuole in rosette leaves during late NS (**d, e**). Vesiculation of the ER and the vacuole in rosette leaves during late NS (**f**_1_**-f**_5_). F1-F5 are individual pictures of a Z-stack through vesiculated ER (**f**_1_ and **f**_2_) and the central vacuole undergoing fragmentation (**f**_2_**-f**_5_, arrows). The integrity of the nuclear envelope is retained at this stage (**f**_5_, empty arrowhead). Bolus formation in cauline leaves during late NS (**g**). Bolus formation in silique walls during late NS (**h**). Bolus formation in rosette leaves of darkened whole plants (**i**). Vesiculation of the ER in rosette leaves during DIS (**j**). The polygonal architecture of the ER is still visible despite strong bolus formation and punctate distribution of fluorescence signals (**g-i**) or vesiculation (**j**). DMP1-eGFP is strongly expressed in the cortex of root tips (**k-o**), in phloem bundles (**p**), and weakly expressed in other cell layers of the root (**q**). In the cortex DMP1-eGFP localizes to the tonoplast, highlighting vacuole biogenesis (**k**, single picture, **m** maximal projection and **i**, light transmission). Magnification of the region near the root tip (**n**) shows multiple vacuoles of different size and shape which tend to form a central vacuole in the root elongation zone (**o**). Subcellular localization in the phloem bundle could not been determined but strong fluorescence signals in structures which might be ER boluses were observed (**p**). ER localization in roots is shown in (**q**, arrow). Scale bar, 10 μm.

To scrutinize whether dual localization in *Arabidopsis* is an artifact by overexpression of DMP1-eGFP by the *CaMV 35S* promoter, we expressed the same fusion protein from the native *DMP1* promoter in transgenic plants. In accordance with the senescence-associated activity of the *DMP1* promoter [1], DMP1-eGFP fluorescence is only detectable in mature, early and late senescing rosette leaves, senescing cauline leaves, senescing silique walls and roots (Figure 7). In mature-to-early senescing rosette leaves, DMP1-eGFP strongly accumulates in the ER and to a lesser extent in the tonoplast. However, the tonoplast signals are hardly distinguishable from the ER signals (Figure 7a). ER bodies are occasionally observed (Figure 7b). Formation of boluses resembling the eponymous protein aggregates reported by Griffing [13] and vesiculation events are observed in rosette leaves (Figure 7c,e,f), cauline leaves (Figure 7g) and silique walls (Figure 7h) undergoing natural senescence. Darkening of single rosette leaves (Figure 7i) or whole plants (Figure 7d and j) lead to similar events. In individual cells disintegration of the ER is discernible (Figure 7c). In these cells the junctions of the ER tubules seem to swell (arrow) and vesiculate (arrowhead). We suggest that bolus formation precedes vesiculation of the ER, though it cannot be excluded that the two processes represent two different fates for cells undergoing senescence. Indeed, neighboring cells of the same type undergoing induced senescence can display different degrees of bolus formation and vesiculation (Additional file 1: Figure S6). In other cells, fragmentation of the tonoplast is obvious (Figure 7d and e, arrows) with occasional persistence of residual ER network (Figure 7e, arrowhead), suggesting a close succession of the two vesiculation processes. Figure 7 f_1_-f_5_ show ER which already underwent vesiculation (arrowheads) and fragmentation/vesiculation of the tonoplast (arrow), indicating that ER breakdown precedes tonoplast breakdown. Tonoplast vesiculation is more rarely observed than ER vesiculation during developmental or dark induced senescence. Tonoplast breakdown is presumably only a short-lived phase as it rapidly and irreversibly leads to cell death. The persistence of the nuclear membrane (Figure 7 f_5_, open arrowhead) in spite of progressed ER breakdown is reminiscent of the events in tobacco during stage 5.

In roots, vacuolar localization of DMP1-eGFP is obvious in the cortex of root tips (Figure 7k-o). In accordance with the current view of vacuole biogenesis, the emerging cells near the root tip contain several vacuoles differing in size (Figure 7d) whereas the older cells in the elongation zone have fewer vacuoles or a single central vacuole (Figure 7o). In these cells the plasma membrane is also labeled (Figure 7n, arrows), which is supposedly due to a truncated isoform of DMP1 (to be published elsewhere). In the phloem bundles, the subcellular localization could not be determined because of the small size of cells (Figure 7p). The ER network was also visible in roots, highlighting once more the ability of DMP1-eGFP to target multiple subcellular membrane systems (Figure 7q).

## Discussion

### DMP1-eGFP shows dual intracellular targeting and induces membrane remodeling

Transient expression of DMP1-eGFP in tobacco epidermis cells revealed dynamic targeting of the protein to the tonoplast and the ER. This may indicate that DMP1 possesses competitive tonoplast targeting and ER retention signals, as has been found in proteins that are dually targeted to different compartments such as mitochondria and chloroplasts [14]. The most striking effect of DMP1-eGFP is the complex remodeling and formation of novel membrane structures at the tonoplast and the ER. Shortly after transfection (stage 1), DMP1-eGFP induces the formation of bulbs resembling those first described in young *Arabidopsis* cotyledons [10]. As they disappear upon progression of cell expansion, formation of these bulbs is believed to be independent of the cytoskeleton [11]. It was initially suggested that they might serve as membrane reservoirs during rapid cell and vacuole expansion [10]. More recently Saito et al. reported that bulbs emerge in germinating seeds by fusion of small vacuoles [15]. Bulbs were found in numerous tissues, at various developmental stages, under stress conditions and in different plant species, suggesting additional functions [15-22]. Specific functions of the bulbs differing from the tonoplast are also indicated in our study by the segregation of DMP1-eGFP and TPK1-mRFP at bulb membranes. A similar case was made by Saito and colleagues who showed that though γ-TIP-GFP and GFP-AtRab7c were both located at the tonoplast, only γ-TIP-GFP was present at the bulbs [10].

In stage 2 DMP1-eGFP mostly localizes in the ER, which undergoes severe reorganization during that stage. As the cortical ER has in stage 1 a tubular morphology and contains almost no DMP1-eGFP, it is likely that during stage 2 the protein induces reorganization of the ER to large cisternae. Similarly, induction of ER cisternae formation has been observed by expressing GFP fused to the transmembrane domain of calnexin [23,24]. Transition from tubular to cisternal architecture of the ER has been reported in response to various abiotic and biotic stresses and presumingly reflects modification in ER functions. The tubule-to-cisternae transition may be correlated to the integrity of the actin cytoskeleton, which precisely overlies the ER network [25], as its disassembly as well as myosin inhibition both lead to loss of the tubular structure and the formation of large cisternae [26].

The DMP1-eGFP-labeled tubules, which appear at the beginning of stage 3, form a network that matches the cortical ER (Figure 4 a-c). Towards the end of stage 3 the DMP1-eGFP-labeled network dissociates from the cortical ER network (Figure 4d-f). In contrast to a differentiation of the ER into distinct subregions with different protein content, e.g. reticulons which accumulate at edges of ER sheets [27,28], we observe a segregation of the DMP1-eGFP-labeled structures from the ER, resulting in two physically disconnected membrane networks (Figure 4d-f and g-j). The DMP1-eGFP-labeled network appears more relaxed and less reticulated than the ER network associated with the YFP-HDEL and RFP-p24 markers. We observe additionally the formation of crystalloid ER which consists exclusively of smooth ER (Figure 5 j_1_ and j_2_) as has already been described in other studies [29-35]. Thus, we propose that the DMP1-eGFP-labeled network consists of smooth ER whereas the network labeled by YFP-HDEL and RFP-p24 represents rough ER. As crystalloid ER has not been described in tobacco epidermis cells before, an expansion of the smooth ER (stage 3) triggered by accumulation of DMP1-eGFP in the cortical ER (stage 2) seems plausible. To our knowledge this is the first documentation of the proliferation of tobacco epidermis cell cortical ER into smooth ER. Although the biological relevance of this observation has yet to be determined, the fluorescent DMP1 fusion protein is a novel *in vivo* indicator for the plasticity and differentiation capacity of the ER.

The transition from stage 3 to 4 is accompanied by the disappearance of the smooth ER network and the accumulation of vacuolar membrane sheets. The resulting foamy phenotype of the vacuole during stage 4 represents a massive increase of the tonoplast surface area, implying the supply of new lipids which are known to be synthetized in the smooth ER. We therefore speculate whether the proliferation of smooth ER reflects an increased synthesis of lipids which eventually accumulate in the tonoplast leading to the foamy phenotype. However, as the DMP1-eGFP-labeled network appears to break down to smaller tubules and vesicles (Figure 4g and k), it is also conceivable that these structures are directly taken up by the vacuole by fusing with the tonoplast leading ultimately to the foamy phenotype. Vacuolar membrane sheets have so far been proposed to be bulbs which lost their spherical shape and adopted a sheet-like configuration [11,20]. This model is supported by our observation that the local separation of DMP1-eGFP and TPK1-mRFP signals in stage 1-bulbs (Figure 3a-d) re-emerges somewhat later in the foamy stage 3-vacuolar sheets (Figure 4e-h_2_). The sponge-like structures observed during late stage 3 and stage 4 may represent residual membrane islands originating from bulbs and vacuolar sheets. Despite severe membrane remodeling, stage 4-cells appear to remain viable for several days, suggesting that the essential physiological functions of the cells are still intact. Stage 5 presumably represents the fate of cells which have passed a developmental point of no return and undergo cell death marked by fragmentation of the vacuole and the ER.

### In *Arabidopsis* DMP1 highlights dynamic restructuring of the ER and vacuole late in developmental and induced senescence

The fate of the ER during senescence is largely unknown. It has been reported to disappear like other organelles during petal senescence [7] and even less is known about its destiny during developmental (NS) or induced leaf senescence (DIS). We discovered that the first morphological alteration during NS and DIS affecting the whole ER is the formation of aggregates termed ‘boluses’. DMP1-eGFP expression by its native, senescence-associated promoter illuminates the formation of boluses in all studied organs undergoing NS or DIS (rosette leaves, cauline leaves and siliques). Bolus formation and ER fragmentation are most prominent in darkened plants, as this treatment probably synchronizes cells and subsequent cell death. Comparable aggregations within the ER have been shown by overexpressing reticulons, a class of ER proteins with membrane curvature-inducing properties, in tobacco epidermis cells. The luminal protein YFP-HDEL displays a punctate repartition within the ER network when coexpressed with RTNLB13 and RNTLB1-4 [28]. It was suggested that overexpression of reticulons induces constrictions of the ER tubules creating luminal pockets in which soluble proteins accumulate. A formation of boluses resembling those in our study was also observed within the lumen and at membranes of the ER subdomain that associates with the chloroplast upon expression of a luminal, YFP-HDEL, and a transmembrane protein, YFP-RHD3 [13]. Our study yields for the first time evidence that bolus formation at the ER network occurs during plant development and concerns the whole ER network within a cell. Bolus formation presumably reflects a restrained protein mobility and motion within the ER as a consequence of fading ER integrity and function during late senescence. The timing of membrane reorganization suggests that the subsequent stage in ER network degradation is a brief vesiculation phase (Figure 7c). The fate of these vesicles is unclear. They are possibly taken up by the vacuole for further degradation.

We only rarely observed fragmentation of the vacuole. It is not clear whether this fragmentation indicates PCD during senescence or another unrelated type of cell death that occurs independently in individual cells. Senescence is classified as ‘autolytic’ or ‘vacuolar’ plant cell death, that is marked by an initial increase of the vacuolar volume by fusion of smaller vacuoles and the shrinkage of the cytoplasm, followed by rupture of the tonoplast and rapid degradation of the cytoplasm [3,36]. Fragmentation of the vacuole has not yet been reported in autolytic cells death. However, in epidermis cells the central vacuole already occupies more than 90 % of the cell volume which precludes the fusion of smaller vacuoles. It is conceivable that in these cells a rupture of the tonoplast is accompanied by a brief fragmentation of the central vacuole that can hardly be visualized in the EM.

### A function of DMP1 in membrane fusion and fission events during development?

The molecular function of DMP1 is still unknown. However, as from stage 2 all phases of membrane remodeling in tobacco cells expressing DMP1-eGFP are associated with membrane fusion or fission, it seems likely that DMP1 is actively involved in these processes. In stage 1, the formation of bulbs results from invagination of the tonoplast forming a double-membrane inside the vacuole [10,20] and may thus not require membrane fusion or fission. During stage 2, ER reorganization from tubular to cisternal elements requires membrane fusion. Apparent segregation of smooth ER from the cortical ER network can only be explained by membrane fission and membrane expansion. The emergence of free tubules and small vesicles in the cytosol that obviously originate from the smooth ER-network requires membrane fission. Formation of vacuolar sheets and foamy membrane structures in stage 4 presumably needs membrane fission and fusion, and eventually vesiculation of the vacuole during stage 5 necessitates membrane fission.

Also in *Arabidopsis* the localization of DMP1-eGFP suggests a close connection to membrane fission/fusion events. In root tips undergoing central vacuole biogenesis, known to take place by fusion of smaller vacuoles and vesicles, DMP1-eGFP is no longer expressed in the cortex layer as soon as the central vacuole is established. This strongly argues for a participation of DMP1 in vacuole biogenesis in this cell layer. During senescence, the protein is associated rather with the reverse reaction, i.e. the fragmentation of the ER and the tonoplast by membrane fission. It is conspicuous that DMP1 shares a similar overall architecture with the reticulons, which have been shown to shape ER tubules by membrane bending [37,38]. The members of both protein families possess four transmembrane domains. In reticulons these are arranged in two long hydrophobic “hairpins” leading to a wedge-like topology with very short loops 1 and 3 and a longer loop 2 facing the cytosol [28]. The DMP proteins have also short loops 1 and 3 and a longer loop 2 [1]. Whether DMP1 is directly, e.g. by enforcing membrane distortion, or indirectly, e.g. by interaction and cooperation with other proteins, responsible for the membrane remodeling phenomena reported in this study remains to be elucidated.

## Conclusions

AtDMP1 is a novel senescence-associated membrane protein that is targeted to the ER and the tonoplast. The DMP1-eGFP fusion protein illuminates dynamic ER and tonoplast remodeling processes and endomembrane reorganization during leaf senescence: (1) Transient expression of DMP1-eGFP in tobacco leaf cells led to temporally ordered remodeling events of the ER (tubules-to-sheets transition, proliferation of smooth ER and formation of crystalloid ER) and the tonoplast (formation of bulbs, vacuolar sheets and “foamy” structures). (2) Stable expression in *Arabidopsis* by the native promoter demonstrated for the first time the occurrence of aggregates inside the ER membranes (“boluses”) and vesiculation of the ER during developmental and induced senescence. (3) In root tips of *Arabidopsis* plants DMP1 is associated with vacuole biogenesis. Comparable temporally ordered restructuring of the ER, the tonoplast or other membranes has not yet been reported for other fusion proteins. We conclude that DMP1 is actively involved in endomembrane remodeling, membrane fission and membrane fusion in senescing cells and in root development.

## Methods

### Generation of constructs

*35S:DMP1-eGFPmRFP-MUB2*[39] and *TPK1-mRFP*[40] expression vectors were generated and modified as described in [1]. *RFP-p24*[41] and *YFP-HDEL* were provided by David Robinson (University of Heidelberg, Germany) and Chris Hawes (Oxford Brookes University, UK), respectively. *DMP1p:DMP1-eGFP* was generated by amplifying a 2364 bp *DMP1promoter:624 bp DMP1* ORF fragment on genomic *Arabidopsis* Col-0 DNA with the primers 5′-CGGTCTAGAGAGAACAAAATCCTCCGTATC-3′ and 5′-AACTGCAGCGGCAGAGACCGAGGCTTTC-3′, followed by digestion of the PCR product with *XbaI/PstI* and ligation into *XbaI/PstI* digested binary vector pGTkan3 [1].

### Plant material, growth conditions and plant transformation

*Arabidopsis thaliana* Col-0 and *Nicotiana benthamiana* plants were grown and transformed as described [1]. All *Agrobacterium* cultures were resuspended to OD_600_ = 0,05 prior to tobacco infiltration. To reduce silencing of the transgenes, all constructs were co-infiltrated with the silencing suppressor p19 [42].

### Confocal microscopy

Confocal microscopy was performed on a Leica TCS-SP5 AOBS (acousto-optical beam splitter) confocal laser scanning microscope (Leica Microsystems) equipped with water immersion objectives (20x with numerical aperture of 0.7 and 63x with numerical aperture of 1.20). Excitation/emission wavelengths were: eGFP: 488 nm (argon laser)/495 nm - 510 nm; YFP: 514 nm (argon laser)/525 nm - 555 nm; mRFP and mCherry: 561 nm (diode-pumped solid-state (DPSS) laser)/585  nm - 655  nm. Multi-color imaging of cells co-expressing eGFP, YFP and mRFP (or mCherry) was performed by sequential scanning to prevent crosstalk. Post-acquisition image processing was performed with the Leica LAS AF software (Leica Microsystems). Depending on the object structure either single pictures or maximum projections resulting from z-stacks are shown. The following pictures result from maximum projections: Figure 1b and c; Figure 2b-e; Figure 3i-k; Figure 4a-c, g-j, k and l; Figure 5a-d, l-o and m; Figure 7m; Additional file 1: Figure S6.

### Transmission electron microscopy

For fixation, substitution and embedding of one mm^2^ leaf sections (see Additional file 1: Table T1 for protocol) a laboratory microwave (PELO BioWave® 34700–230, Ted Pella, Inc., Redding CA, USA) was used. For analysis in a Tecnai G2 Sphera transmission electron microscope (FEI Company, Eindhoven, Netherlands) at 120 kV, ~70 nm ultra thin sections were cut with a diamond knife and contrasted with uranyl acetate and lead citrate prior to examination.

## Authors’ contributions

AK carried out all experiments, performed data analysis and interpretation, prepared the figures and wrote the manuscript. MM carried out the transmission electron microscopy. RK planned the study and wrote the manuscript. All authors read and approved the final manuscript.

## Supplementary Material

Additional file 1 **Compilation of Additional file 1:** Figure S1 – S6 and Additional file 1: Table T1. Additional file 1: Figure S1: DMP1-eGFP does not localize to the ER during stage 1. Additional file 1: Figure S2: Tight association between DMP1-eGFP-labeled tubules and vacuolar sheets. Additional file 1: Figure S3: The vacuolar sheets are double membranes. Additional file 1: Figure S4: Exclusion of TPK1-mRFP at contact zones within foamy membrane structures. Additional file 1: Figure S5: The sponge-like structures are tonoplast domains. Additional file 1: Figure S6: Bolus formation and vesiculation of the ER occur asynchronously within a tissue. Click here for file
