# Supplementary material for: Arabidopsis senescence-associated protein DMP1 is involved in membrane remodeling of the ER and tonoplast
Source: BMC Plant Biol. 2012 Apr 24;12:54. doi: 10.1186/1471-2229-12-54 (PMC3438137; doi:10.1186/1471-2229-12-54)
Supplement: Additional file 1 — Compilation of Additional file 1: Figure S1 – S6 and Additional file 1: Table T1. Additional file 1: Figure S1: DMP1-eGFP does not localize to the ER during stage 1. Additional file 1: Figure S2: Tight association between DMP1-eGFP-labeled tubules and vacuolar sheets. Additional file 1: Figure S3: The vacuolar sheets are double membranes. Additional file 1: Figure S4: Exclusion of TPK1-mRFP at contact zones within foamy membrane structures. Additional file 1: Figure S5: The sponge-like structures are tonoplast domains. Additional file 1: Figure S6: Bolus formation and vesiculation of the ER occur asynchronously within a tissue. [file 1471-2229-12-54-S1.pdf]

Additional Table T1

Fixation, substitution and embedding solutions for transmission electron microscopy.

**Process**	**Chemical**	**Power [W]**	**Time**	**Vacuum [mm Hg]**
Primary fixation	2.0% (v/v) glutaraldehyde and 2.0% (w/v) formaldehyde in 50 mM cacodylate buffer	150<br>0<br>150<br>0<br>150<br>0	1 min<br>1 min<br>1 min<br>1 min<br>1 min<br>1 min	15<br>15<br>15<br>15<br>15<br>15
Wash	1x with 50 mM cacodylate buffer (pH 7.3) and 2x aqua dest.	150<br>0	45 sec<br>45 sec	0<br>0
Secondary fixation	1% (v/v) osmiumtetroxide in aqua dest.	0<br>80<br>0<br>80	1 min<br>2 min<br>1 min<br>2 min	15<br>15<br>15<br>15
Wash	2 x aqua dest.<br>1 x aqua dest.	150<br>0	45 sec<br>15 min	0<br>0
Dehydration	Ethanol series: 30%, 40%, 50%, 60%, 75%, 90%, 2x 100% and 1 x Propylenoxide	150	45 sec	0
Resin infiltration	Spurr resin in propylenoxide: 25%, 50%, 75%, 2x 100%<br>1 x 100% Spurr resin	250<br>--	3 min<br>1 h	0<br>--
Polymerisation	70°C in a heating cabinet	--	24 h	--

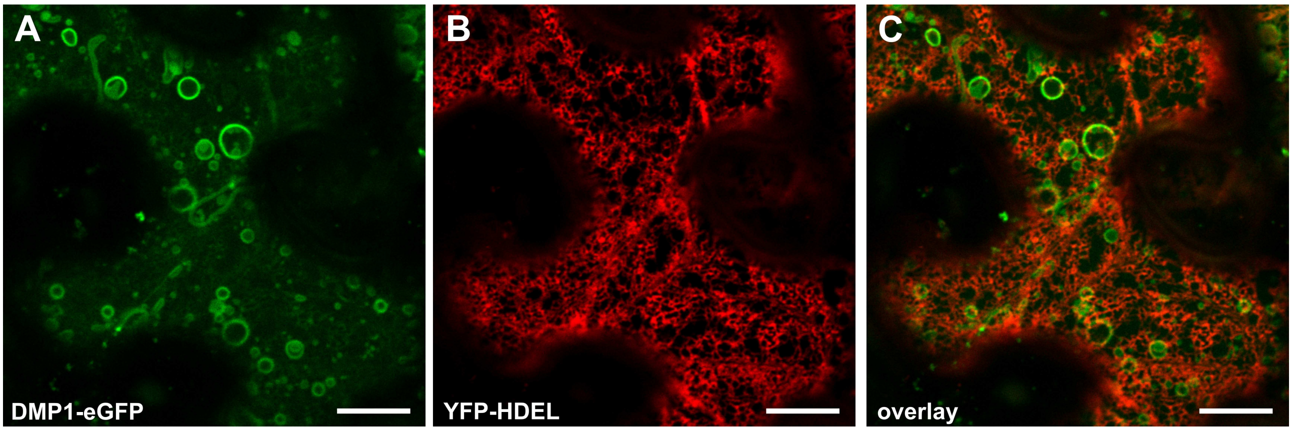

Additional Figure S1 - DMP1-eGFP does not localize to the ER during stage 1. Coexpression of DMP1-eGFP (A) and YFP-HDEL (B) labeling the lumen of the ER shows separation of the fluorescence signals (C). In some cells hardly discernible DMP1-eGFP signals show up which presumably are non-specific background (A). Scale bar, 10 µm

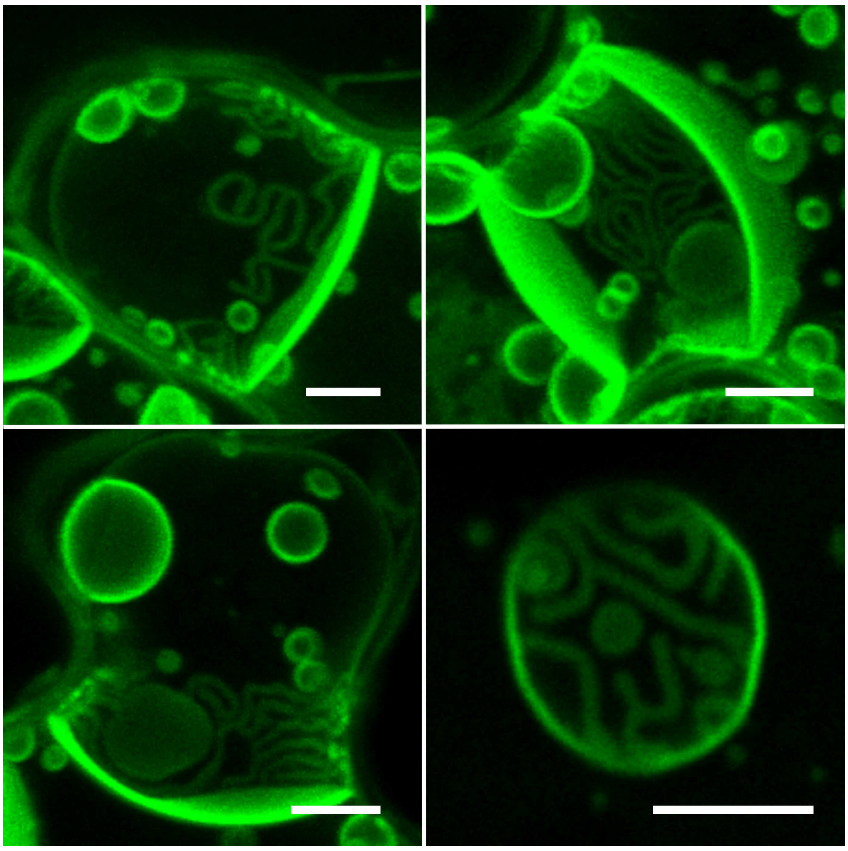

Additional Figure S2 - Tight association between DMP1-eGFP-labeled tubules and vacuolar sheets. DMP1-eGFP labeled tubules appear occasionally in tight association with vacuolar sheets, suggesting vacuolar uptake of the tubules. Scale bar, 5 µm

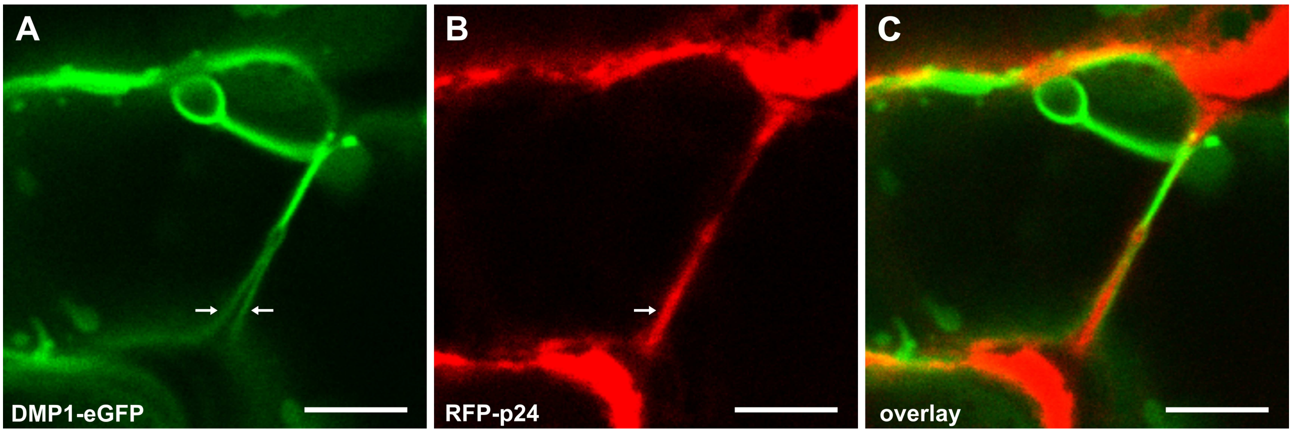

Additional Figure S3 - The vacuolar sheets are double membranes. Coexpression of DMP1-eGFP (A) and RFP-p24 (B) shows ER squeezed between the two membranes (C) forming a vacuolar sheet (arrows). Scale bar, 20 µm

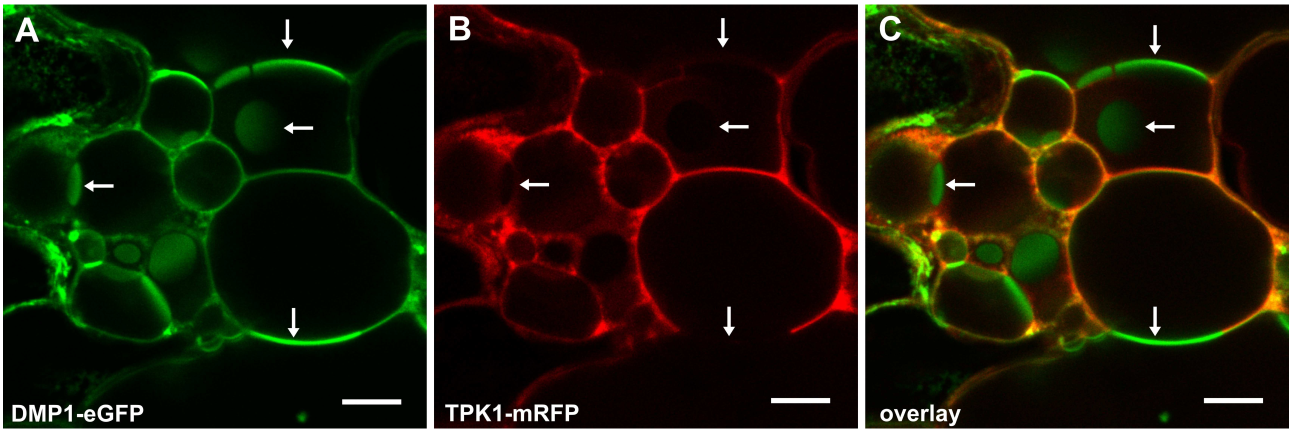

Additional Figure S4 - Exclusion of TPK1-mRFP at contact zones within foamy membrane structures. In areas where DMP1-eGFP strongly accumulates (A) TPK1-mRFP is frequently excluded, indicating inhomogeneous membrane composition. The signals appear to exclude each another (C, arrows). These areas are often round shaped and found at contact zones of adjacent sheets (A-C, arrows). Scale bar, 10 µm

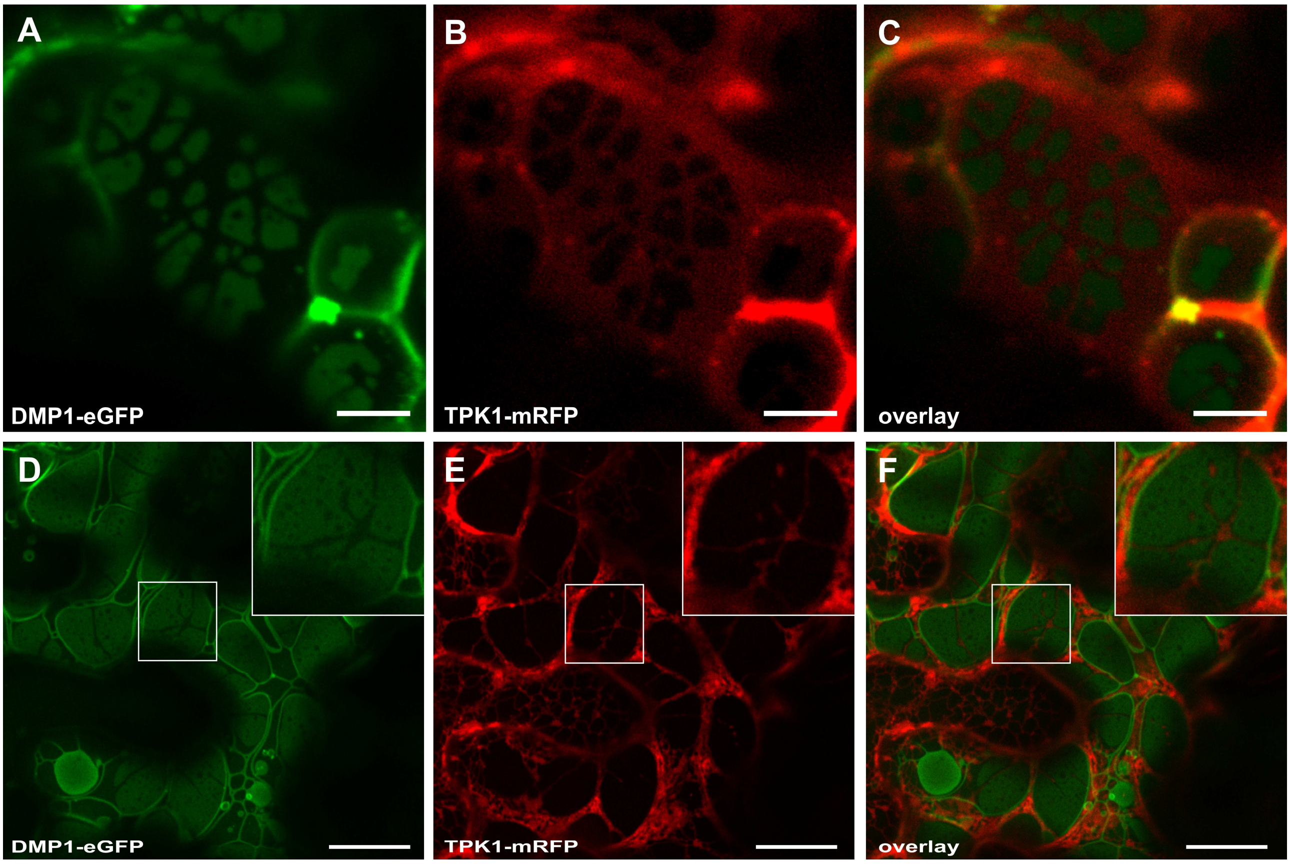

Additional Figure S5 - The sponge-like structures are tonoplast domains. Coexpression of DMP1-eGFP (A) and TPK1-mRFP (B) demonstrates separation of the fluorescence signals during stages 3 and 4 (C). The signals appear at the same confocal plane, indicating tonoplast areas with different membrane properties. Occasionally, sponge-like structures extend throughout the whole cell (D). In these cells, the ER labeled with RFP-p24 (E) as well as the tubular network labeled with DMP1-eGFP (A) appear to be compacted in spaces lacking sponge-like structures (F) suggesting spatial proximity between the tonoplast and the plasma membrane. Scale bar, A-C, 5 µm; D-F, 20 µm

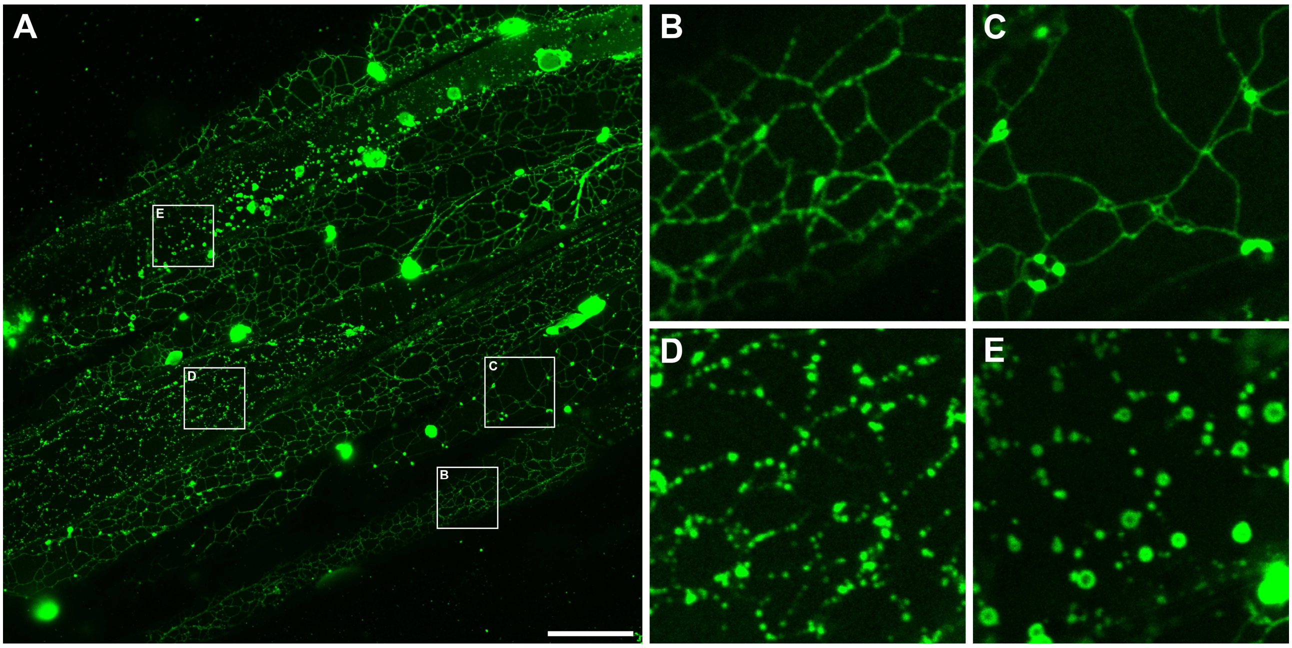

Additional Figure S6 - Bolus formation and vesiculation of the ER occur asynchronously within a tissue. Epidermis cells along a leaf vein of a rosette leaf after darkening of the whole plant for 7 days (A). Magnification of individual cells from panel A (B-E). ER network showing low background bolus formation (B). ER network with apparent loss of reticulation and boluses concentrated at tubule junctions (C). Intense bolus formation with the fluorescence signals exhibiting a punctate distribution (D). Vesiculation of the entire ER network (E). Scale bar, 30 µm
